# 

**Published:** 1867-09

**Authors:** 


### CONTENTS.

### ORIGINAL COMMUNICATIONS.

PAGE.

ARTICLE I.—Valedictory Address on behalf of the Faculty to the Graduating Class in Atlanta Medical College, at the Annual Commencement, on Friday, August 30, 1867. By J. M. Johnson, M. D. .... 287

ARTICLE II.—Gun-shot Wound of the Neck—Injury of the Spinal Cord. By Stith Malone, A. M., M. D., of Athens, Ala. .... 298

ARTICLE III.—Diptheria. By Reuben Searcy, M. D., Tuscaloosa, Ala. .... 300

ARTICLE IV.—Extracts from Minutes of Atlanta Medical Society .... 301

### SELECTIONS.

Inflammation of the Uterus .... 305

Novelties and Improvements in Medicine .... 312

Innervation of the Heart .... 318

Medullary Sarcoma of the Spleen .... 321

An Anodyne Formula .... 322

### EDITORIAL AND MISCELLANEOUS.

Atlanta Medical College .... 323

To Our Patrons .... 324

Our Journal .... 325

Dr. Yandell's Letter .... 326

Vomiting of Pregnancy .... 327

New Anæsthetic .... 328

Application of Collodion in Cholera .... 328

